# New avenues for understanding what deep networks learn from EEG

**DOI:** 10.3389/frobt.2025.1625732

**Published:** 2025-10-09

**Authors:** Robin T. Schirrmeister, Tonio Ball

**Affiliations:** 1 Medical Physics, Department of Diagnostic and Interventional Radiology, Medical Center—University of Freiburg, Faculty of Medicine, University of Freiburg, Freiburg, Germany; 2 Neuromedical A.I. Lab, Department of Neurosurgery, Medical Center—University of Freiburg, Faculty of Medicine, University of Freiburg, Freiburg, Germany; 3 BrainLinks-BrainTools, IMBIT (Institute for Machine-Brain Interfacing Technology), University of Freiburg, Freiburg im Breisgau, Germany

**Keywords:** electroencephalogram (EEG), brain-signal decoding, medical AI, interpretable deep learning, pathology decoding

## Abstract

An important but unresolved question in deep learning for EEG decoding is which features neural networks learn to solve the task. Prior interpretability studies have mainly explained individual predictions, analyzed the use of established EEG features, or examined subnetworks of larger models. In contrast, we apply interpretability methods to uncover features learned by the complete network. Specifically, we introduce two complementary architectures with dedicated visualization techniques to obtain an approximate understanding of the full network trained on binary classification into nonpathological and pathological EEG. First, we use invertible networks—networks that are designed to be invertible—to generate prototypical input signals for each class. Second, we design a very compact network that is fully visualizable, while still retaining reasonable decoding performance. Through these visualizations, we find both expected features like higher-amplitude oscillations in the delta and theta frequency bands in the temporal region for the pathological class as well as surprising differences in the very low sub-delta frequencies below 0.5 Hz. Closer investigation reveals higher spectral amplitudes for the healthy class at the frontal sensors in these sub-delta frequencies, an unexpected feature that the proposed visualizations helped identify. Overall, the study shows the potential of visualizations to understand the network prediction function without relying on specific predefined features.

## 1 Introduction

Interpretability is an important aspect of deep learning on medical data. A wide range of methods have been proposed to explain deep networks in this context, ranging from local methods, which aim to explain individual predictions (e.g., saliency maps, perturbation-based techniques), to global methods, which aim to capture the features learned by the network as a whole (e.g., concept activation vectors (van der Velden et al., 2022; Tjoa and Guan, 2021). There remains debate about the appropriate use of such methods in the medical domain, particularly regarding their suitablity for generating trust in individual predictions (Ghassemi et al., 2021; Reddy, 2022). Nevertheless, there is broad agreement that improved understanding of the features learned by deep networks can provide value, for example, by helping to discover novel biomarkers or by revealing reliance on inappropriate shortcuts (Ghassemi et al., 2021; Reddy, 2022).

For decoding pathologies from EEG recordings, interpretability methods can reveal some of the learned EEG features. Studies used local interpretability methods like Shapley Values, Grad-CAM (Selvaraju et al., 2017) or layerwise relevance propagation (Bach et al., 2015) to explain individual predictions (de Bardeci et al., 2021; Vahid et al., 2019; Jemal et al., 2022; Uyttenhove et al., 2020). Other studies used methods like deep dream to explain a part of the network like an individual neuron (Dubreuil-Vall et al., 2020; Zhang et al., 2020). Some studies also designed networks to make some part of the network interpretable (Jemal et al., 2022; Salami et al., 2022). Finally, some studies tried global interpretability methods to show what the network learned about the relationship between well-known features like spectral power (e.g., in the alpha/beta band) and the class labels (Schirrmeister et al., 2017; Gemein et al., 2020).

A gap remains in global, feature-agnostic interpretability methods for EEG, i.e., approaches that visualize the prediction function of a network without relying on predefined features or specific input examples. Such methods can reveal learned EEG features beyond established markers and may facilitate the discovery of novel features. However, explaining the prediction function of a trained network with complete faithfulness is likely impossible. Human-understandable explanations of large deep networks can at best approximate the true prediction function, inevitably sacrificing some faithfulness. By contrast, smaller and more compact networks may be explained more faithfully, though often at the cost of reduced decoding performance. In the following, we describe two types of networks with corresponding interpretability methods, each balancing faithfulness and expressivity in different ways.

In this study, we introduce two EEG decoding architectures that enhance interpretability by either producing class prototypes or enabling full-network visualization, and we apply them to EEG-based diagnosis. First, we adapt an invertible network, i.e., a deep network that is invertible by design, for EEG decoding. We train this invertible network, termed *EEG-InvNet*, as a generative classifier and visualize prototype signals for each class and each electrode, thereby providing a compressed representation of each class directly in the raw input space. Second, we propose a highly compact network, termed *EEG-CosNet*, in which the entire architecture can be visualized. We train it to mimic the prediction function of the invertible network. Its parameters can be fully visualized as scalp topographies and temporal signal patterns, providing an interpretable representation of the learned mapping from signals to classes.

Visualizations of the invertible networks revealed both well-established EEG features, such as temporal slowing and occipital alpha, as well as unexpected patterns in the sub-delta frequency range ( ≤0.5 Hz). Visualizations of the EEG-CosNet showed regular oscillatory patterns in the alpha- and beta-band associated with healthy recordings, alongside a diverse set of slower or more irregular waveforms linked to pathological recordings. In the sub-delta range, the visualizations further revealed a frontal component predictive of the healthy class and temporal components predictive of the pathological class. Manual inspection of the sub-delta range confirmed lower amplitudes for the pathological class, supporting the utility of our visualization methods as hypothesis-generating tools.

## 2 Methods

**FIGURE 1:**
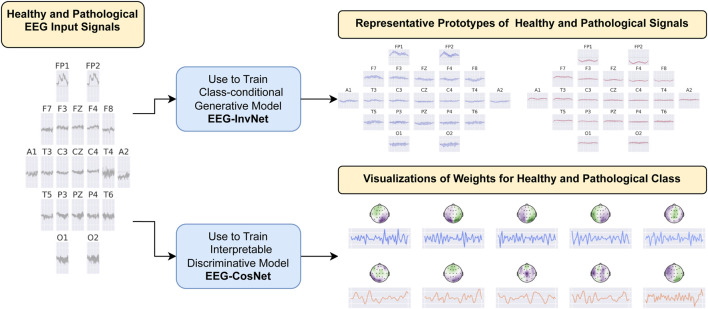
Overview of the interpretability approaches. We developed two methods for investigating EEG features learned by neural networks in the task of pathology decoding. In the first approach, we train a class-conditional generative invertible network, *EEG-InvNet*, and use it to generate prototype EEG signals for the healthy and pathological classes. In the second approach, we train a compact, interpretable discriminative network, *EEG-CosNet*, and visualize its learned discriminative weights for the two classes.

We developed two interpretability approaches for analyzing EEG features learned by neural networks: one based on invertible networks trained as generative models, and another based on compact, interpretable networks trained as discriminative models. An overview of both approaches is provided in Figure 1, with detailed descriptions in the following subsections.

### 2.1 Invertible networks

Invertible networks are neural networks composed of layers that are explicitly designed to be invertible, meaning the input can be exactly reconstructed from the output. Several types of invertible layers exist; one of the most widely used is the coupling layer (Kingma and Dhariwal, 2018). + A coupling layer splits a multidimensional input vector x into two disjoint subsets, $x_{1}$ and $x_{2}$ . It then uses $x_{2}$ to compute an invertible transformation of $x_{1}$ , while leaving $x_{2}$ unchanged. Concretely, for an additive coupling layer, the forward computation is:

$$\begin{array}{cc} y_{1} & =x_{1}+f\left(x_{2}\right)\\ y_{2} & =x_{2} \end{array}$$

and the inverse computation is:

$$\begin{array}{cc} x_{1} & =y_{1}-f\left(y_{2}\right)\\ x_{2} & =y_{2} \end{array}$$

For splitting the dimensions of a time series, one may, for example, define $x_{1}$ as the mean and $x_{2}$ as the difference between two neighboring samples, analogous to one stage of a Haar wavelet transform. The function f is typically implemented by a neural network; in our case, it is realized as a small convolutional network. Additional invertible layers used in this work include activation normalization layers, which scale and shift channel activations, and invertible linear layers, which mix channels linearly using an invertible weight matrix W , as described by Kingma and Dhariwal (2018).

#### 2.1.1 Training as generative models

Invertible networks can be trained as generative models by maximizing the average log-likelihood of the training data. In this setting, the network is optimized to maximize the average log-probability log⁡p(x) of the training inputs x Theis et al. (2016). + Invertible networks assign probabilities to inputs x by mapping them to a latent representation z=f(x) and evaluating their density under a predefined prior distribution $p_{z}\left(z\right)$ in that latent space (see Theis et al. (2016) for details).

#### 2.1.2 Training as classifiers

Invertible networks trained as class-conditional generative models can also serve directly as classifiers. This can be implemented, for example, by assigning a separate prior distribution in the latent space to each class. Given the class-conditional probability densities $p\left(x|c_{i}\right)$ , the posterior class probabilities can be obtained via Bayes’ theorem as (assuming uniform prior class probabilities):

$$p\left(c_{i}|x\right)=\frac{p\left(x|c_{i}\right)}{\sum_{j}p\left(x|c_{j}\right)}$$

Purely class-conditional generative training can sometimes yield networks that perform poorly as classifiers (Theis et al., 2016). One proposed explanation is that the optimal average log-likelihood is only marginally higher for class-conditional models compared to class-independent models—on the order of just one bit in the case of binary classification. This difference is much smaller than the variability in log-likelihood typically observed between two independent runs of the same network trained on high-dimensional inputs without class labels (Theis et al., 2016). Although class-conditional models may achieve larger likelihood gains in practice, it is not *a priori* clear whether these improvements translate into better classification performance.

Various methods have been proposed to improve the performance of generative classifiers. For example, prior work has either fixed the per-class latent Gaussian priors to retain equal distances throughout training (Izmailov et al., 2020), or augmented the objective with a classification loss term to the training loss (Ardizzone et al., 2020):

$$\begin{array}{cc} L_{\text{class}}\left(x,c_{i}\right) & =-\log p\left(c_{i}|x\right)\\ & =-\log\frac{p\left(x|c_{i}\right)}{\sum_{j}p\left(x|c_{j}\right)}\\ & =-\log\frac{\exp\left(\log p\left(x|c_{i}\right)\right)}{\sum_{j}\exp\left(\log p\left(x|c_{j}\right)\right)}\\ & =-\log\left(softmax\left(\log p\left(x|c_{i}\right)\right)\right). \end{array}$$

In our work, we experimented with adding such a classification loss term to the training objective, and additionally found that introducing a learned temperature parameter before the softmax stabilized training, leading to:

$$\begin{array}{cc} L_{\text{class}}\left(x,c_{i},t\right) & =-\log\frac{\exp\;\left(\frac{\log p\left(x|c_{i}\right)}{t}\right)}{\underset{j}{\sum }\exp\;\left(\log p\left(x|c_{j}\right)t\right)}\\ & =-\log\left(softmax\;\left(\frac{\log p\left(x|c_{i}\right)}{t}\right)\right). \end{array}$$

Our overall training loss is a weighted sum of the generative loss and the classification loss:

$$\begin{array}{cc} L\left(x,c_{i},t\right) & =L_{\text{class}}\left(x,c_{i},t\right)+L_{\text{gen}}\left(x,c_{i}\right)\\ & =-\log\left(softmax\;\left(\frac{\log p\left(x|c_{i}\right)}{t}\right)\right)-\alpha logp\left(x|c_{i}\right), \end{array}$$

where we set α to the inverse of the input dimensionality, i.e.,

$$\alpha =\frac{1}{dim\left(x\right)}.$$

### 2.2 Invertible network for EEG decoding

**FIGURE 2:**
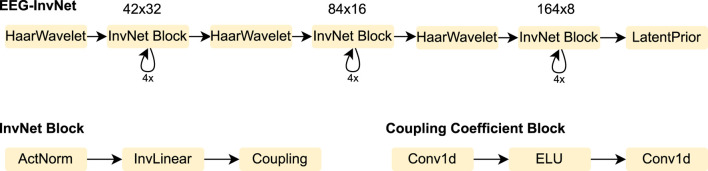
EEG-InvNet architecture. Our EEG-InvNet architecture consists of three stages operating at progressively lower temporal resolutions. The input comprises 2 seconds of EEG from 21 electrodes sampled at 64 Hz, yielding an input of size 21×128 . The data are downsampled using Haar wavelets to sizes 42×32 , 84×16 , and 164×8 across the three stages. Each stage consists of four blocks, with each block containing an activation normalization layer, an invertible linear layer, and a coupling layer. The activation normalization and invertible linear layers operate on the channel dimension, applying the same transformation across all channels at each time point of the feature map. The coupling layer consists of two convolutional layers with an exponential linear unit (ELU) activation in between.

We designed an invertible network, termed *EEG-InvNet*, for EEG decoding, primarily based on invertible components from the Glow architecture (Kingma and Dhariwal, 2018). Our architecture consists of three stages operating at progressively lower temporal resolutions. Similar to Glow, each stage is composed of multiple blocks, each containing an activation normalization layer, an invertible linear channel transformation, and a coupling layer (see Figure 2). Between stages, the temporal signal is downsampled by computing the mean and difference of two neighboring time points and transferring these into the channel dimension. Unlike Glow, all dimensions are processed throughout every stage, and we found this design to achieve competitive accuracy on pathology decoding. We use one Gaussian distribution per class in the latent space. We experimented with both affine and additive coupling layers, but report results using additive layers, as their reduced expressiveness makes them easier to interpret.

### 2.3 Class prototypes

**FIGURE 3:**
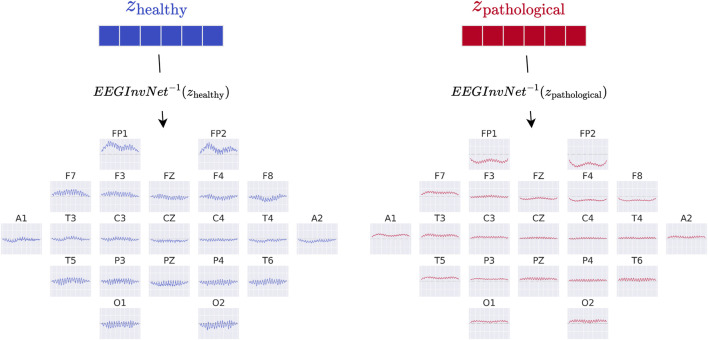
EEG-InvNet class prototypes. Class prototypes are generated by inverting the means, $z_{\text{healthy}}$ and $z_{\text{pathological}}$ , of the per-class Gaussian distributions using *EEG-InvNet*.

In our first visualization, we show the inputs resulting from inverting the means of the gaussian distributions for each class (see Figure 3). For example, the healthy-class prototype $x_{\text{healthy}}$ is obtained by inverting the Gaussian mean $z_{\text{healthy}}$ using the invertible network *EEG-InvNet*:

$$x_{\text{healthy}}={EEG-InvNet}^{-1}\left(z_{\text{healthy}}\right).$$

These visualizations can be interpreted as prototype examples of each class. However, individual features within a prototype should be interpreted with caution. For example, if a prototype contains a prominent alpha-band oscillation at one electrode, this does not imply that the oscillation is independently predictive of the class, since other features may also contribute. Nevertheless, such prototypes can already suggest potential discriminative features for further investigation.

### 2.4 Per-electrode prototypes

**FIGURE 4:**
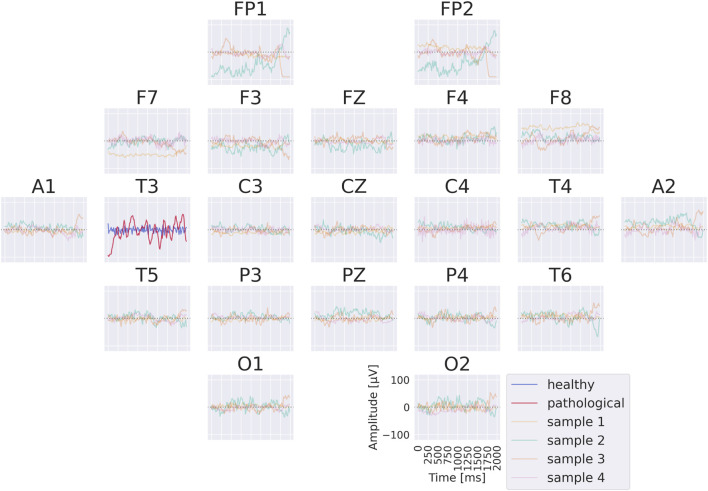
EEG-InvNet per-electrode class prototypes. Per-electrode prototypes are obtained by optimizing synthetic class-specific signals for a single electrode to yield high predicted class probabilities, irrespective of the signals at other electrodes. Signals at the remaining electrodes are sampled from the training data. In the example, prototypes for electrode T3 are learned for both the healthy and pathological classes, with four sampled signals shown for the remaining electrodes. In practice, a much larger number of samples is used. Class probabilities are marginalized over the non-optimized channels, as described in the text.

One way to obtain more interpretable prototypes is to synthesize them on a per-electrode basis. Specifically, we synthesize a signal $x_{e_{k}}^{*}$ for a given electrode $e_{k}$ such that the predicted probability of a target class $c_{i}$ is high, irrespective of the signals at the other electrodes (see Figure 4). For electrode $e_{k}$ and class $c_{i}$ , we optimize $x_{e_{k}}^{*}$ by maximizing the marginal likelihood (generative loss):

$$p\left(x_{e_{k}}^{*}|c_{i}\right)=\int p\left(x|c_{i};x_{e_{k}}=x_{e_{k}}^{*}\right)\;dx$$

and simultaneously maximizing the classification objective:

$$p\left(c_{i}|x_{e_{k}}^{*}\right)=\frac{p\left(x_{e_{k}}^{*}|c_{i}\right)}{\sum_{j}p\left(x_{e_{k}}^{*}|c_{j}\right)}.$$

In practice, this marginalization is approximated by Monte Carlo sampling: we draw n samples $x_{l}$ from the training distribution and replace the value at electrode $e_{k}$ with the optimized signal $x_{e_{k}}^{*}$ , yielding

$$p\left(x_{e_{k}}^{*}|c_{i}\right)\approx \frac{1}{n}\overset{n}{\underset{l=1}{\sum }}p\left(x_{l}|c_{i};\;x_{l,e_{k}}=x_{e_{k}}^{*}\right).$$

Although only a coarse approximation, this procedure already yields insightful visualizations. For the classification loss, when computing $p\left(c_{i}|x_{e_{k}}^{*}\right)$ , we found it beneficial to divide the log-probabilities $\log p\left(x_{l}|c_{i};\;x_{l,e_{k}}=x_{e_{k}}^{*}\right)$ by the learned temperature parameter t of the classifier:

$$\log p_{\text{clf}}\left(x_{e_{k}}^{*}|c_{i}\right)=logsumexp\;\left(\frac{\log p\left(x_{l}|c_{i};\;x_{l,e_{k}}=x_{e_{k}}^{*}\right)}{t}\right).$$

Without this scaling, the sum $\sum_{l}p\left(x_{l}|c_{i};\;x_{l,e_{k}}=x_{e_{k}}^{*}\right)$ may be dominated by only a few samples when computing $p\left(c_{i}|x_{e_{k}}^{*}\right)$ . This adjustment is applied only to the classification loss $p\left(c_{i}|x_{e_{k}}^{*}\right)$ , not to the generative loss $p\left(x_{e_{k}}^{*}|c_{i}\right)$ .

### 2.5 EEG-CosNet

**FIGURE 5:**
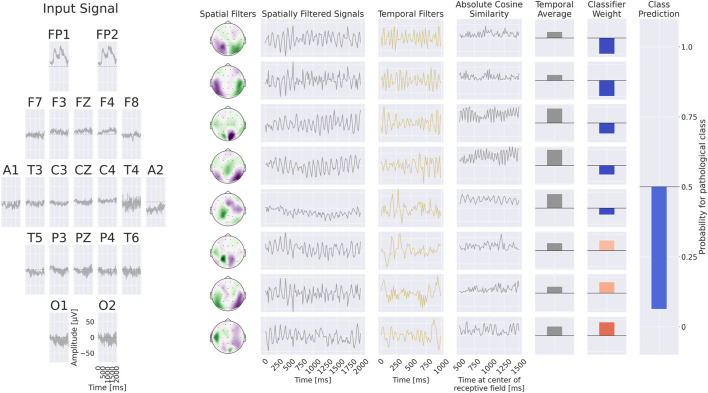
+Example processing of the *EEG-CosNet*. An example EEG input is shown on the left, followed by the processing steps on the right: spatial filtering, absolute cosine similarity with temporal filters, temporal averaging, and final weighting with linear classifier weights for class prediction. In this visualization, the *EEG-CosNet* is configured with only 8 filters; in later experiments we use 64 filters.

Finally, we implemented a compact convolutional network, termed *EEG-CosNet*, which was explicitly designed to be directly interpretable. We distilled the trained *EEG-InvNet* into *EEG-CosNet* by training the latter with the class probabilities predicted by *EEG-InvNet* as soft targets for the classification loss $L_{\text{class}}$ . The *EEG-CosNet* consists of only three steps (see Figure 5 for an example computation):

$$\begin{array}{lll} Spatial\;\mathrm{fi}ltering & & \\ h_{1}=W_{s}^{⊤}x & & \text{Apply spatial filter weights}\;W_{s}\;\text{to the input}\;x\\ Feature\;construction & & \\ h_{2}\left(t\right)=\left|cos\text{\_}sim\left(h_{1}\left[t:t+L\right],f\right)\right| & & \text{Absolute moving cosine similarity with temporal filters}\;f\\ h_{3}=\frac{1}{n_{\text{time}}}\underset{t}{\sum }h_{2}\left(t\right) & & \text{Average over time points in the trial}\\ Classi\mathrm{fi}cation & & \\ h_{4}=W_{c}^{⊤}h_{3} & & \text{Apply classifier weights}\;W_{c}\;\text{to features}\\ p\left(c_{\text{path}}|h_{4}\right)=\sigma \left(h_{4}\right) & & \text{Compute sigmoid }\sigma \left(\cdot \right)\;\text{to get pathological probability} \end{array}$$

Steps 1 and 2 produce spatiotemporal patterns that can be visualized both as temporal waveforms and as scalp topographies, which are subsequently weighted by the linear classifier for the respective classes. We employed cosine similarity to ensure that high output values correspond to spatially filtered signals closely resembling the respective temporal filter. To enhance interpretability, the spatial filter weights and linear classifier weights can be transformed into generative patterns by multiplying them with the electrode covariance (or the averaged absolute cosine similarities) after training; see Haufe et al. (2014) for a detailed discussion of this approach. Importantly, we only apply this covariance transformation to the spatial filters themselves, and do not multiply by the inverse covariance of the filtered signals. This is because each spatial filter is paired with its own temporal filter and should therefore be analyzed independently of the other spatial filters. In our experiments, we employ 64 spatiotemporal filters, each with a temporal length of 64 samples (corresponding to one second at 64 Hz).

### 2.6 Dataset

We evaluate our EEG-InvNet on pathology decoding using a reduced version of the Temple University Hospital Abnormal Corpus (TUAB) (López de Diego, 2017; Harati et al., 2014; Obeid and Picone, 2016). TUAB is a large corpus of clinical EEG recordings, each labeled as either non-pathological or pathological based on accompanying medical reports. The dataset includes recordings acquired at the Temple University Hospital Department of Neurology between 2002 and 2017, covering a wide range of pathologies such as epilepsy, stroke, Alzheimer’s disease, and others. Each recording contains approximately 20 min of EEG data, acquired from at least 21 standard electrode positions with a minimum sampling rate of 250 Hz using a 16-bit A/D converter. TUAB consists of 2,993 recordings (1,521 non-pathological and 1,472 pathological). The dataset creators defined an official into 2,717 training recordings and 276 evaluation recordings, which we adopt to ensure comparability with prior work. To obtain a reduced dataset with cleaner signals, we applied the following preprocessing steps: (i) remove the first minute of each recording, which often contains artifacts; (ii) extract the 2 min immediately following; (iii) downsample the signals to 64 Hz; and (iv) segment the data into 2-s windows, which serve as input examples for the invertible network. This reduced dataset enables faster experimentation while retaining sufficient information for accurate decoding.

### 2.7 Training details

We trained the models using the AdamW optimizer (Loshchilov and Hutter, 2019) cosine annealing with restarts (Loshchilov and Hutter, 2017) every 25 epochs as our learning rate schedule. These hyperparameter settings were not extensively tuned for maximum decoding accuracy. Instead, they were selected to ensure stable training to obtain a model with robust decoding accuracy that can provide insights into discriminative learned features.

## 3 Results

### 3.1 EEG-InvNet decoding results

As shown in Table 1, our proposed EEG-InvNet achieves decoding accuracy comparable to, and in some cases exceeding, that of conventional convolutional neural networks (ConvNets). This competitive performance motivates a deeper investigation into the features learned by the model. Specifically, EEG-InvNet attains an accuracy of 85.5%, outperforming EEGNet as well as both the Deep and Shallow ConvNet baselines, while being slightly below the Temporal Convolutional Network (TCN). These results, which are close to the current state of the art on TUAB, further motivate an analysis of the features learned by EEG-InvNet.

**TABLE 1:** Accuracy of EEG-InvNet on pathology decoding. Accuracies of regular ConvNets taken from Gemein et al. (2020).

Deep	Shallow	TCN	EEGNet	EEG-InvNet
84.6	84.1	86.2	83.4	85.5

### 3.2 Class prototypes

**FIGURE 6:**
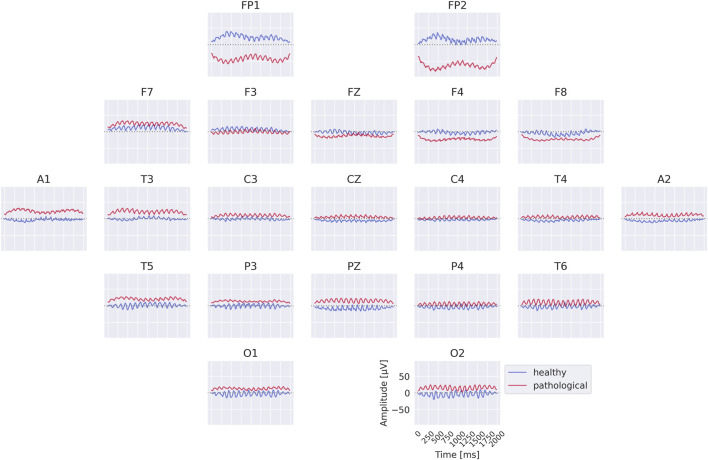
Learned class prototypes from EEG-InvNet. Class prototypes are generated by inverting the learned means of the class-conditional Gaussian distributions from latent space back into input space using the EEG-InvNet trained for pathology decoding (see text for details). The prototypes reveal distinct oscillatory patterns, including alpha oscillations that differ between the two classes. For the healthy class, alpha activity shows larger amplitudes over occipital electrodes, particularly O1, compared to other sites. Interestingly, clear mean differences are apparent at frontal electrodes, highlighting unexpected spatial patterns that distinguish the two classes.

The class prototypes reveal well-known oscillatory features and surprisingly suggest that the invertible network makes use of very-low-frequency information. We visualized these prototypes by inverting the learned latent means of the class-conditional Gaussian distributions (healthy and pathological) back into input space, thereby obtaining the most likely examples under the learned distribution (see also Section 2.3). The visualizations in Figure 6 highlight differences in the alpha rhythm, such as a stronger alpha oscillation at electrode O1 in the healthy prototype. Additional oscillatory differences are visible across both classes, indicating that the prototypes capture a variety of temporal dynamics beyond the alpha band. Surprisingly, the prototypes also differ in the very-low-frequency (sub-delta, ≤ 0.5 Hz) range, with clear differences in mean values at electrodes FP1 and FP2 between the two classes. These findings are examined in more detail in later analyses. Given the caveats of interpreting individual electrode patterns (see Section 2.3), we next turn to per-electrode prototypes for a more localized analysis.

### 3.3 Per-electrode prototypes

**FIGURE 7:**
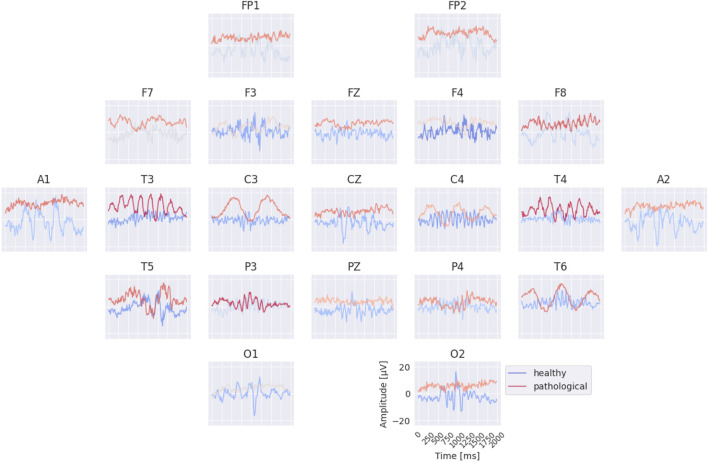
Learned per-electrode prototypes from EEG-InvNet. For each electrode, the input signal was optimized independently to increase the network’s prediction for a given class, while signals at the other electrodes were randomly sampled from the training set. The color scale indicates the average softmax class probability, computed across over 10,000 sampled signals for the non-optimized electrodes. Prominent low-frequency patterns (sub-delta, ≤ 0.5 Hz) are visible for the pathological class at multiple electrode locations.

The per-electrode prototypes reveal distinct features learned for the two classes (see Figure 7). Pathological prototypes show large-amplitude low-frequency oscillations, for example, at T3 and T4, consistent with the well-known biomarker of temporal slowing in pathology. In contrast, healthy prototypes frequently display alpha-band activity, such as at C4 and T6. We also again observe differences in the sub-delta range ( ≤ 0.5 Hz), for instance in the mean values at FP1 and FP2 between healthy and pathological prototypes. Importantly, for several electrodes it was not possible to synthesize a signal that is clearly class-indicative independent of activity at other electrodes. This suggests that the EEG-InvNet did not learn strong class-predictive electrode-specific features at those electrodes.

### 3.4 EEG-CosNet

Results for the EEG-CosNet demonstrate that a large fraction of the predictions made by the invertible network can be recovered from a relatively small set of neurophysiologically plausible spatio-temporal patterns. EEG-CosNet reproduces 88.8% of the EEG-InvNet’s predictions and achieves a test-set label accuracy of 82.6% (see Table 2). This shows that from just 64 spatiotemporal features, the EEG-CosNet is able to predict the vast majority of the EEG-InvNet predictions. However, the remaining performance gap suggests that EEG-InvNet relies on additional features or interactions that EEG-CosNet’s compact architecture cannot fully represent.

**TABLE 2:** Accuracy of EEG-CosNet on labels from EEG-Invnet predictions and original labels.

Split	EEG-InvNet labels	Original labels
Train	92.5	89.1
Test	88.8	82.6

**FIGURE 8:**
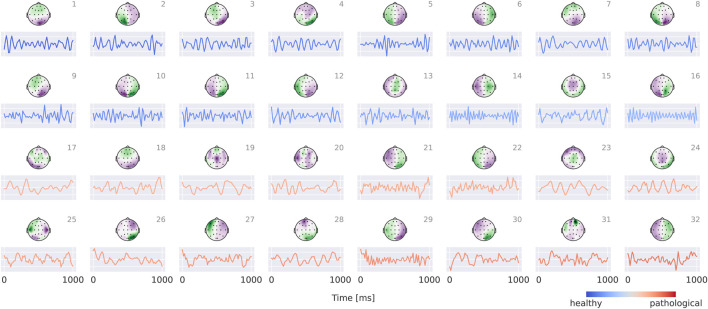
Visualization of the interpretable EEG-CosNet trained to mimic the EEG-InvNet. Scalp plots display the spatial filter weights after transformation into interpretable patterns, and the corresponding convolutional temporal filters are shown below each scalp plot. We display the 16 filters most strongly associated with the healthy class and the 16 most strongly associated with the pathological class (see Supplementary Material for the full network). The color coding of the temporal signals represents the linear classifier weights, transformed to patterns (see Section 2.5 for details). Filters are ordered according to these classifier weights. Note that the polarity of scalp plots and temporal filters is arbitrary, since absolute cosine similarity is applied to the spatially filtered and temporally convolved signals.

Visualizations in Figure 8 reveal that the healthy class is characterized by more regular oscillatory waveforms, particularly in the alpha and beta frequency ranges, whereas the pathological class is associated with waveforms in other frequency ranges and less regular temporal patterns. For instance, in the healthy class, plots 1–4 exhibit oscillations with a pronounced alpha component, while plots 14 and 16 display strong beta components. In contrast, the pathological class shows slower oscillations (e.g., plots 23 and 24) as well as more irregular waveforms (e.g., plots 19 and 30).

### 3.5 Relative power spectra for comparison

**FIGURE 9:**
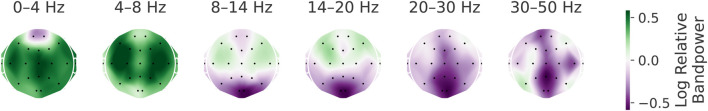
Visualization of relative log-bandpowers. Scalp plots of the logarithm of the relative bandpower between pathological and healthy signals across different frequency bands. Note that the spatial patterns are consistent with the findings from EEG-InvNet and EEG-CosNet visualizations.

To further validate the visualization results, we performed a manual analysis of relative power spectral densities. Specifically, we computed power spectra from 10-s windows with 5-s overlap for both pathological and healthy signals, applying a Hamming window prior to the Fourier transformation. For each electrode, we then calculated the median power across windows in each frequency bin, and finally averaged the results within standard frequency bands: delta (0–4 Hz), theta (4–8 Hz), alpha (8–14 Hz), low beta (14–20 Hz), high beta (20–30 Hz), and low gamma (30–50 Hz). The resulting maps (Figure 9) show patterns consistent with the EEG-InvNet and EEG-CosNet visualizations. Importantly, we included very-low-frequency activity in the delta band to enable comparison with our finding of discriminative information in sub-delta ranges, a phenomenon not highlighted by prior visualizations in the literature (Gemein et al., 2020; Schirrmeister et al., 2017).

## 4 Investigation of sub-delta frequencies

One surprising observation from the visualizations is the difference in sub-delta frequency components ( ≤0.5 Hz) between the two class prototypes. For example, the substantially different mean amplitudes in the prototypes at electrodes FP1 and FP2 suggest that very low-frequency activity differs between the two classes at these sites. However, given the inherent limitations of interpreting class prototypes, one cannot be certain about the precise relationships between EEG activity and class membership solely from these plots. Nevertheless, these observed differences motivated a more detailed investigation of the sub-delta frequency range.

To assess the role of very low frequencies, we trained an EEG-InvNet on data low-pass filtered to retain only frequencies below 0.5 Hz. Specifically, we removed all Fourier components above 0.5 Hz from each full recording as well as from each 2-s input window provided to the network. The EEG-InvNet achieved 75.4% accuracy under this condition, indicating that even very low-frequency components remain fairly informative about the pathological status of the recordings. We additionally trained an EEG-CosNet with a temporal filter spanning the entire 2-s input window and found it to reach 75.0% test accuracy. Finally, we trained an 8-component Gaussian mixture model (Fourier-GMM) in the Fourier domain. For each electrode, only three features were retained: the real part of the 0-Hz component (corresponding to the summed amplitude of the input window) and the real and imaginary parts of the 0.5-Hz Fourier component. Each of the eight mixture components was associated with learnable class weights that determined its contribution to the class-conditional distribution. The Fourier-GMM also achieved 75.4% test accuracy. All results are shown in Table 3.

**TABLE 3:** Test accuracy on data lowpassed below 0.5 Hz.

EEG-InvNet	EEG-CosNet	Fourier-GMM
75.4	75.0	75.4

### 4.1 EEG-InvNet visualizations

**FIGURE 10:**
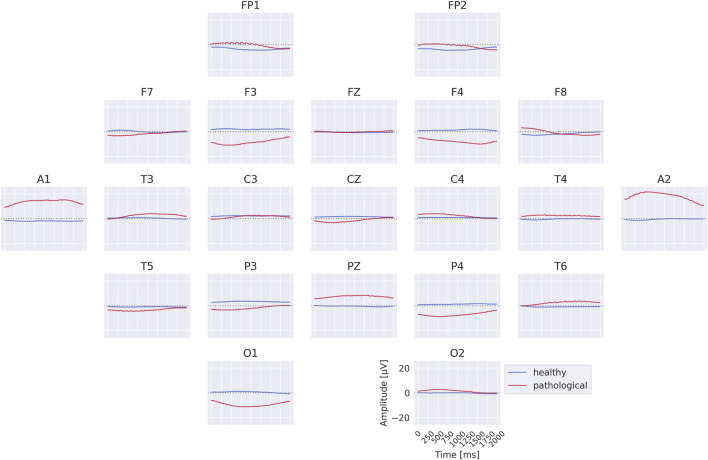
Class prototypes for the EEG-InvNet trained on data lowpassed to be below 0.5 Hz. Note large differences at A1 and A2.

**FIGURE 11:**
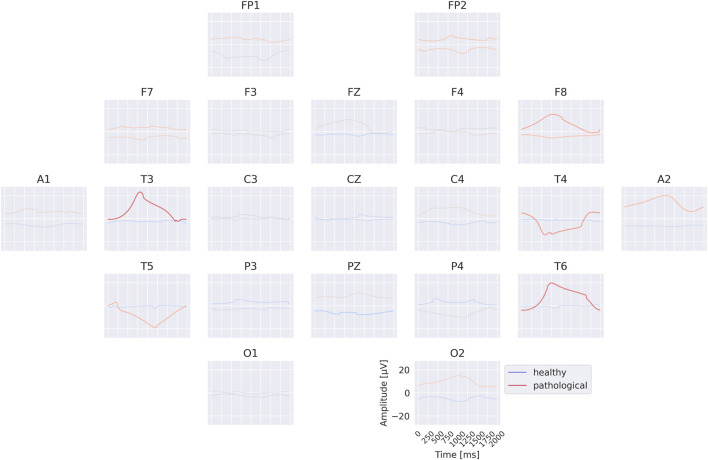
Per-electrode prototypes for EEG-InvNet trained on data lowpassed below 0.5 Hz. Note strongly predictive signals at T3, T4, T6.

The visualizations of the EEG-InvNet reveal several differences between the two classes. The class prototypes in Figure 10 exhibit distinct signal patterns across most electrodes, with particularly pronounced differences at A1 and A2. The per-electrode prototypes in Figure 11 highlight strong differences at electrodes T3, T4, and T6. Overall, these visualizations suggest that a range of low-frequency differences may contribute to class discrimination, motivating further analyses to identify the most relevant features.

### 4.2 EEG-CosNet visualizations

**FIGURE 12:**
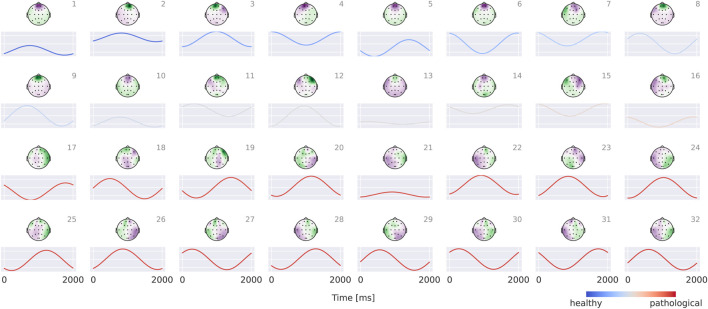
Spatiotemporal patterns for EEG-CosNet trained on lowpassed data below 0.5 Hz. Note large frontal components associated with healthy class.

The visualization of the EEG-CosNet in Figure 12 reveals strong frontal components associated with the healthy class and temporal components associated with the pathological class. The temporal components are consistent with the per-electrode visualization, and the frontal components were already apparent as differences in mean signal values in the class prototypes of the original data. These visualizations more clearly highlight specific features as strongly discriminative between the two classes.

### 4.3 Fourier-GMM visualizations

**FIGURE 13:**
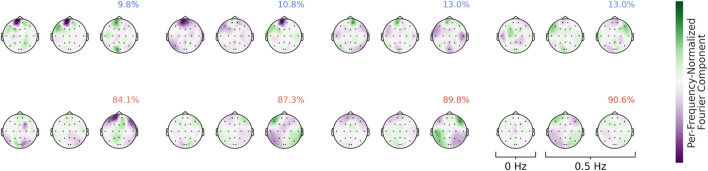
Means of the Fourier-GMM mixture components in the Fourier domain. Scalp plots are shown for the 0-Hz bin as well as the real and imaginary parts of the 0.5-Hz bin. Mixture components are ordered according to their pathological class weights, which are also indicated as colored text in the top-right corner of each plot. Colormaps are scaled separately for each frequency bin. Strong frontal patterns are evident in the mixture components associated with the healthy class.

Visualizations of the Fourier-GMM in Figure 13 again reveal frontal components associated with the healthy class, as well as components with spatial topographies involving temporal regions that are associated with the pathological class. Overall, the visualizations consistently indicate a frontal component predictive of the healthy class and additional components with spatial topographies often encompassing temporal and adjacent regions that are predictive of the pathological class. In the following, we further manually validate the unexpected frontal component.

### 4.4 Spectral analysis

**FIGURE 14:**
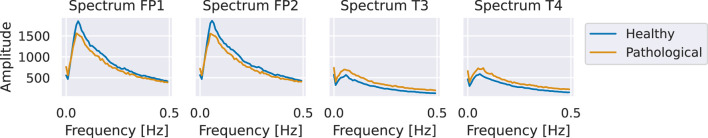
Average amplitudes of sub-delta frequencies at frontal and temporal electrodes. The figure shows class-wise averages of the amplitudes of Fourier-transformed training inputs. The healthy class exhibits higher amplitudes at frontal electrodes and lower amplitudes at temporal electrodes compared to the pathological class.

We validated the patterns identified in our visualizations using spectral analysis. Specifically, we computed the class-wise averages of the amplitudes of the Fourier-transformed training inputs. We found that the healthy class exhibited larger amplitudes at the frontal electrodes, whereas the pathological class showed larger amplitudes at the temporal electrodes (see Figure 14). We emphasize that this manual spectral analysis of the sub-delta frequencies was motivated by the visualization findings and would otherwise have been unlikely to be conducted.

## 5 Discussion

We introduced two approaches that combine neural networks with visualization methods for learned EEG features, and applied them to the task of pathology diagnosis. The first approach employs invertible networks to generate prototypical signals for each class, while the second approach leverages a compact, interpretable network in which all parameters can be directly visualized. Both approaches provide visualizations of what the networks have learned in the input space.

Class prototypes can serve as hypothesis generators for potentially discriminative features, including unexpected ones. These prototypes are visualized in the input space, which allows arbitrary features to be revealed. However, they are challenging to interpret, as they present only a single prototypical example per class and require additional reasoning to identify relevant features within these examples. Thus, their primary role is to generate hypotheses about potentially discriminative features, which must then be analyzed further. Their value in this work is demonstrated by highlighting unexpected discriminative information in the sub-delta frequency range, which we subsequently validated through manual spectral analysis.

We also introduced a per-electrode variant of the prototypes, designed to be more easily interpretable. In this approach, we optimize a prototypical signal at a single electrode, associated with one class, independently of the signals at other electrodes. This variant can reveal only single-electrode features, such as large oscillations at specific frequencies, but not multi-electrode features, such as phase-locking across electrodes. This restriction facilitates interpretability and revealed neurophysiologically plausible patterns, such as slow oscillatory activity at temporal electrodes associated with pathology.

Both types of prototypes reveal complementary aspects of the features learned by the trained network. The overall prototypes can capture arbitrary combinations of features, but they are more challenging to interpret. In contrast, the per-electrode prototypes are restricted to single-electrode features, which makes them easier to interpret. Together, these methods highlight different but complementary aspects of the learned features.

As our final visualization method, we introduced a compact and interpretable network, EEG-CosNet, in which all parameters can be directly visualized. This addresses the limitation of prototypes, which may only reveal parts of the learned features. *A priori*, it is not clear whether such a restricted and compact network can achieve competitive performance on pathology decoding. The visualizations reveal a variety of predominantly oscillatory waveforms: more regular oscillations in the alpha and beta frequency ranges associated with the healthy class, and less regular, lower-frequency oscillations associated with the pathological class. This suggests that such features are sufficient to yield reasonable decoding accuracies for pathology.

One intriguing finding suggested by our visualizations was the decreased power at frontal electrodes in the sub-delta frequency range ( ≤ 0.5 Hz) for the pathological class. This feature was revealed by the prototypical signals, which exhibited unexpected differences in the sub-delta range. It was subsequently confirmed through manual spectral analysis, thereby validating the value of the visualizations as hypothesis generators for learned features. To our knowledge, this feature has not been previously described in relation to pathological EEG. One potential explanation may be a reduction of eye movements due to impaired neuromuscular eye control in pathological patients; however, further research is required to better understand this phenomenon.

The features learned in this study both confirmed previously reported findings and uncovered novel ones. The presence of alpha oscillations associated with the healthy class and lower-frequency oscillations associated with the pathological class are consistent with prior findings in the literature (Gemein et al., 2020; Schirrmeister et al., 2017). In contrast, the differences observed in the sub-delta frequency range have not been reported in similar visualizations before (Gemein et al., 2020; Schirrmeister et al., 2017).

In future work, the interpretability work here could be extended to better capture intra-class variations. For example, the class prototypes could be extended by generating multiple complementary subprototypes that reveal complementary discriminative information. Similarly, the single compact interpretable network can be replaced by several small networks in a mixture-of-experts framework.

Overall, the visualization methods developed in this work provide an insightful avenue for advancing the understanding of pathological features learned by deep neural networks from EEG recordings.

## Data Availability

The original contributions presented in the study are included in the article/Supplementary Material, further inquiries can be directed to the corresponding author.
