# Supplementary material for: New avenues for understanding what deep networks learn from EEG
Source: Front Robot AI. 2025 Oct 9;12:1625732. doi: 10.3389/frobt.2025.1625732 (PMC12545007; doi:10.3389/frobt.2025.1625732)
Supplement: Supplementary file 1 [file Image1.pdf]

Supplementary Material

1 SUPPLEMENTARY FIGURE

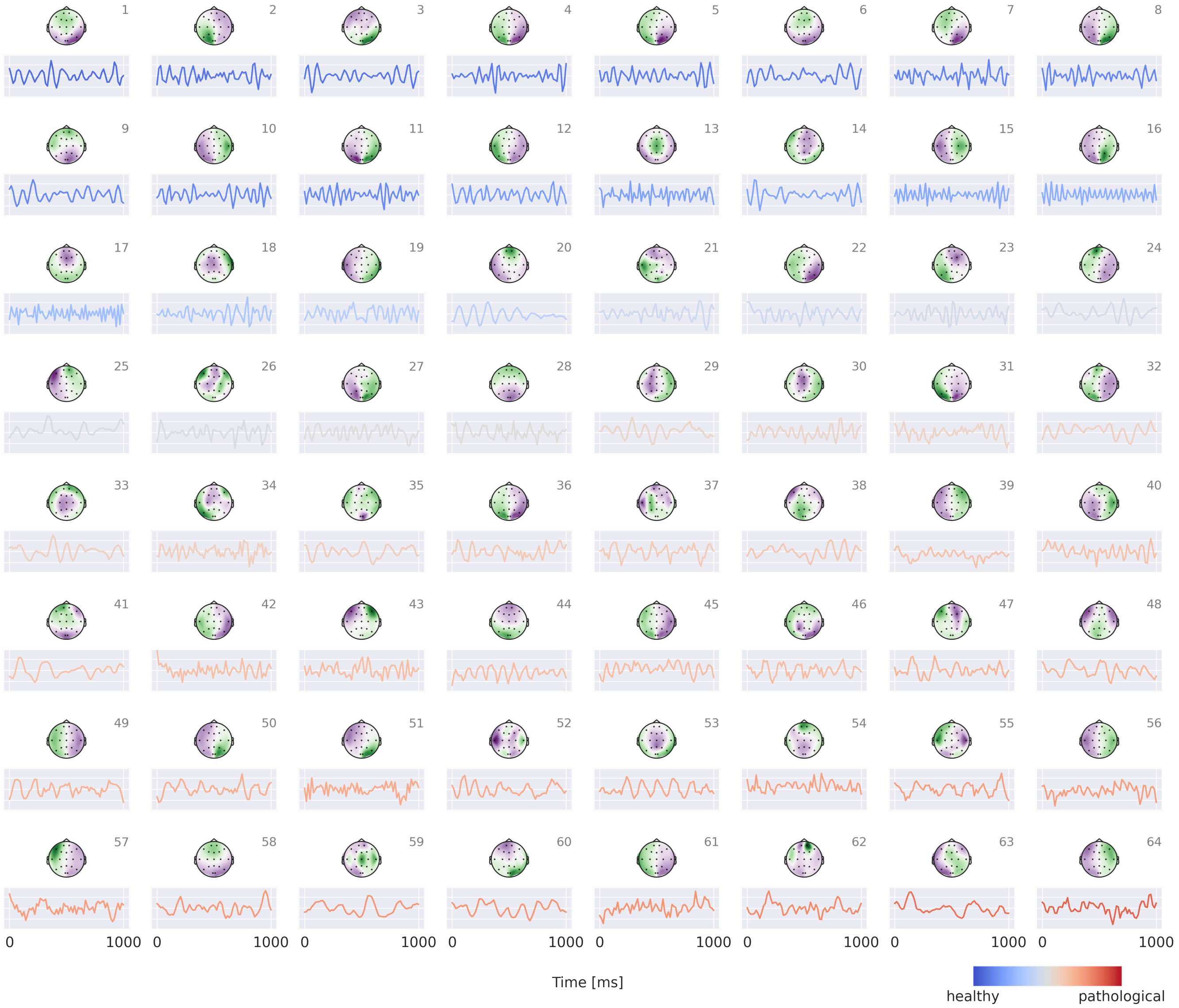

Figure S1. Visualization of small interpretable EEG-CosNet trained to mimic the EEG-InvNet. Scalp Plots are spatial filter weights transformed to patterns, signals below each scalp plot show the corresponding convolutional filter. Signal colors represent the weights of the linear classification layer, transformed to patterns (see Section 2.5 in the main manuscript for an explanation). Plots are sorted by these colors. Note that polarities of the scalp plots and temporal waveforms are arbitrary as absolute cosine similarities are computed on the spatially filtered and temporally convolved signals.
